# Association between weight-adjusted waist index and arterial stiffness in hypertensive patients: The China H-type hypertension registry study

**DOI:** 10.3389/fendo.2023.1134065

**Published:** 2023-03-17

**Authors:** Yurong Xiong, Weidong Shi, Xiao Huang, Chao Yu, Wei Zhou, Huihui Bao, Xiaoshu Cheng

**Affiliations:** ^1^ Department of Cardiovascular Medicine, The Second Affiliated Hospital of Nanchang University, Nanchang, Jiangxi, China; ^2^ Jiangxi Provincial Cardiovascular Disease Clinical Medical Research Center, Nanchang, Jiangxi, China; ^3^ Jiangxi Sub-Center of National Clinical Research Center for Cardiovascular Diseases, Nanchang, China; ^4^ Department of Cardiovascular Medicine, Wuyuan People’s Hospital, Shangrao, Jiangxi, China; ^5^ Center for Prevention and Treatment of Cardiovascular Diseases, The Second Affiliated Hospital of Nanchang University, Nanchang, Jiangxi, China

**Keywords:** weight-adjusted waist index, arterial stiffness, BMI, hypertension, obesity

## Abstract

**Objective:**

Exploring the relationship between (weight-adjusted waist index) WWI and arterial stiffness (AS) in the total and different BMI populations among patients with hypertension.

**Methods:**

This study enrolled 5232 hypertensive subjects, a subset of the China H-type Hypertension Registry Study. WWI was calculated as WC (cm) divided by the square root of weight (kg). Brachial-ankle pulse wave velocity (baPWV) was measured to determine AS.

**Results:**

The mean WWI was 10.97 (0.78)cm/√kg. In multiple logistic analyses showed that there were significant dose-dependent association between WWI with baPWV in a dose-dependent manner in total population (β 57.98, 95% CI 44.06-71.90), and in different BMI group: group 1 (BMI<18.5kg/m^2^) (β 94.30, 95% CI 39.36-149.23), group 2 (18.5-23.9kg/m^2^) (β 74.21, 95% CI 54.57-93.85), group 3 (≥24kg/m^2^) (β 26.11, 95% CI 5.22-47.01). In stratified analysis, stronger associations between WWI and baPWV were observed in patients with higher BP or lower BMI. Sensitivity analysis by excluding patients treated with lipid-lowering agents did not change the association between WWI and baPWV.

**Conclusion:**

For hypertensive patients, we found that WWI was positively associated with baPWV in different BMI groups. WWI might be considered as an intervening factor in preventing and treatment of AS, besides BP management.

## Introduction

Arterial stiffness (AS) is an independent predictor of cardiovascular diseases (CVD) and mortality (1, 2). Brachia-ankle pulse wave velocity (baPWV) measurement is commonly used to assess arterial wall stiffness in epidemiological studies, as it is accurate, simple and non-invasive. Recently, increasing numbers of studies have revealed that baPWV is positively related to future risk of CVD events (3), diabetes (4), and all-cause mortality (5).

As generally known, obesity is closely associated with AS and can independently predict the future risk of AS (6–8). Several studies have shown that weight loss achieved through lifestyle measures can improve AS (9, 10). Moreover, existing studies show that muscle indices are negatively related to the risk of AS (11). Body mass index (BMI) is the major measure of obesity and is widely used in many epidemiological studies because of its low cost, simplicity, and availability. Nevertheless, current researches are still controversial about the relationship between BMI and AS among different populations. In the past decade, some studies have found a positive association between BMI and AS (12–14), while others have found a negative association (15, 16), and others have found no association (17, 18). These discrepancies between the different studies may be attributed to different sample sizes, ethnic and regional variations. Moreover, BMI is not able to discriminate between muscle and fat. Weight-adjusted waist index (WWI) was first proposed in 2018 as a new anthropometric index for predicting the risk of CVD events and mortality (19). Moreover, a study from Korea shows that elevated WWI is closely related to high body fat and low muscle mass (20). Draw a question, whether WWI would better identify the risk of AS compared to BMI.

Hypertension is a significant risk factor for the development of AS (21). According to China Hypertension Survey (2012-2015), the prevalence of hypertension among Chinese adults is 23.2% (22). Obesity is a proven risk factor for hypertension (23). The co-existence of obesity and hypertension significantly increases the risk of developing AS. According to a cohort study of 10338 subjects, WWI is closed associated with future risk of hypertension (24). However, the ability of WWI for assessing the risk of AS among the hypertensive population is poorly understood. Therefore, this study aimed to explore the association between WWI and AS among hypertensive patients in the total population and different BMI populations. In addition, we further assess the mediating roles of blood pressure (BP) satisfaction on the association between WWI and AS.

## Methods

### Study participants

Our study participants came from China H-type Hypertension Registry Study that previously reported (25, 26) (Registration number: ChiCTR1800017274). In brief, this is a real-world, multicenter, observational registry study carried out in southern China from March 2018 to August 2018. Inclusion was carried out in participants with hypertension aged 18 years and older. Hypertension was defined as seated, resting BP≥140/90mmHg at the screening, self-report, or undergoing anti-hypertensive treatment. The exclusion criteria included neurological abnormalities, inability to follow up according to the study protocol, or plans to relocate shortly, and the patients, who were not suitable for inclusion or for long-term follow-up assessed by study physicians. All participants provided written informed consent. The protocol was approved by the Ethics Committee of Institute of Bio-medicine, Anhui Medical University (NO. CH1059) and the Second Affiliated Hospital of Nanchang University (NO. 2018019).

Our study was conducted on a subset of 5233 subjects with complete baPWV data from China H-type Hypertension Registry Study. After excluding participants with loss of WWI data (n =1), 5232 participants were included in the final analysis, as shown in **Figure S1**.

### Assessment of covariates

According to a standard operating procedure, all subjects were interviewed by a trained study coordinator. The information related to age, sex (male or female), smoking, drinking, physical activity (mild, moderate or vigorous), and medical history (diabetes mellitus, hyperlipidemia, duration of hypertension and current medication usage) were abstracted from a standard questionnaire. The heights, weights, and waist circumference (WC) of all patients were collected by trained researchers according to the standard process. BMI was calculated as weight (kg) divided by the square of height (m^2^). BMI was defined as underweight (<18.5kg/m^2^), normal (18.5-23.9kg/m^2^), overweight or obesity (≥24kg/m^2^) according to the cut-off point for Chinese adults (27). WWI (cm/√kg) was calculated as WC (cm) divided by the square root of weight (kg). Resting blood pressure (BP) was measured by the automated electronic device (Omron; Dalian, China) in a standardized manner.

After a 10 h fasting period, blood samples were obtained from all subjects. The blood samples would be measured in Biaojia Biotechnology Laboratory, Shenzhen, China. Fasting plasma glucose (FPG), fasting lipids (total cholesterol, high-density lipoprotein-cholesterol (HDL-C), low-density lipoprotein cholesterol (LDL-C), and triglycerides) and homocysteine(Hcy) were determined using automatic clinical analyzers (Beckman Coulter).

### BaPWV measurements

The baPWV was assessed with Omron Colin BP-203RPE III device (Omron Health Care, Kyoto, Japan) following the recommended standard procedures. The specific details of baPWV measurement were described previously (26).

### Statistical analysis

Baseline characteristics are shown as mean ± standard deviation (SD) for continuous variables, and as n (%) for categorical variables. Descriptive analyses were conducted according to WWI quartiles using t-test or Chi-square tests to compare between-group differences. Dose-response association of WWI with baPWV was assessed using a generalized additive model (GAM) and a fitted smoothing curve (penalized spline method). We used a multivariate linear regression model (beta coefficient [β] and 95% confidence interval [CI]) to assess the relationship between WWI and baPWV in the total and different BMI populations by controlling the confounders in three models. Model 1: adjusted for age; Model 2: Model 1 plus sex, current smoking, current drinking, physical activity, BMI; Model 3: Model 2 plus systolic blood pressure (SBP), diastolic blood pressure (DBP), duration of hypertension, diabetes mellitus, hyperlipidemia, antihypertensive agents, antidiabetes agents, lipid-lowering agents, FPG, Hcy, triglyceride, HDL-C, and LDL-C. The general linear model was determined to compare the difference of baPWV in total and different BMI populations. Subgroup and stratified analyses were also done. A sensitivity analysis was also conducted. In sensitivity analyses, the same analyses were performed after excluding patients treated with lipid-lowering agents. In addition, To determine whether the association between WWI and baPWV was mediated by BP (SBP or DBP), a simple mediation analysis was completed (**Figures S2A, B**).

All data analyzed were using the statistical package R (http://www.r-project.org) and Stata software, version 14.0 (StataCorp). A 2-tailed P < 0.05 was considered to be statistically significant.

## Results

### Characteristics of the subjects

Overall, 5232 hypertensive subjects were enrolled in the final analysis (2603 men and 2629 women, aged 64.7 ± 9.5 years). The mean WWI was 10.97 (0.78) cm/√kg. Mean baPWV was 1858.4 (420.7) mm/s. **Table 1** shows the characteristics of the patients according to the WWI quartiles. The participants with higher WWI had higher values of age, weight, WC, BMI, SBP, duration of hypertension, diabetes mellitus, antihypertensive agents, antidiabetes agents, FBG, and LDL-C. In addition, higher WWI level was negatively associated with lower values in male, smoking, drinking, physical activity, Hcy, and HDL-C.

**Table 1:** Clinical characteristics of the study population.

	Weight-adjusted waist index, cm/√kg				*P* value
	Q1 (<10.5)	Q2 (≥10.5, <10.9)	Q3 (≥10.9, <11.5)	Q4 (≥11.5)	
N	1308	1308	1308	1308	
Age, y	63.2 ± 9.8	63.3 ± 9.8	64.6 ± 9.1	67.5 ± 8.8	<0.001
Male, n (%)	887 (67.8)	791 (60.4)	615 (47.0)	311 (23.8)	<0.001
Weight, kg	73.7 (7.7)	81.6 (7.8)	85.2 (7.3)	88.5 (8.3)	<0.001
WC, cm	54.6 (10.3)	58.5 (11.0)	58.4 (9.9)	55.4 (10.6)	<0.001
BMI, kg/m^2^	21.4 ± 3.2	23.3 ± 3.3	24.0 ± 3.1	24.4 ± 3.6	<0.001
Smoking, n (%)	753 (57.6)	690 (52.8)	587 (44.9)	417 (31.9)	<0.001
Drinking, n (%)	592 (45.3)	541 (41.4)	454 (34.7)	320 (24.5)	<0.001
Physical activity, n (%)					<0.001
Mild	643 (49.2)	717 (54.8)	733 (56.0)	796 (60.9)	
Moderate	319 (24.4)	310 (23.7)	307 (23.5)	241 (18.4)	
Vigorous	346 (26.5)	281 (21.5)	268 (20.5)	271 (20.7)	
SBP, mmHg	145.2 ± 17.9	146.5 ± 17.9	147.4 ± 17.2	149.2 ± 17.3	<0.001
DBP, mmHg	88.7 ± 10.9	89.5 ± 11.4	89.0 ± 10.6	87.2 ± 11.0	<0.001
Duration of hypertension, y	4.0 (2.0-9.0)	5.0 (2.0-10.0)	5.0 (2.0-10.0)	6.0 (2.0-10.0)	<0.001
Diabetes mellitus, n (%)	178 (13.6)	215 (16.4)	287 (21.9)	305 (23.3)	<0.001
Hyperlipidemia, n (%)	185 (14.1)	265 (20.3)	260 (19.9)	230 (17.6)	<0.001
Antihypertensive agents, n (%)	760 (58.1)	799 (61.1)	815 (62.3)	833 (63.7)	0.025
Antidiabetes agents, n (%)	34 (2.6)	53 (4.1)	83 (6.3)	69 (5.3)	<0.001
Lipid-lowering agents, n (%)	39 (3.0)	42 (3.2)	51 (3.9)	49 (3.7)	0.530
Homocysteine, umol/L	19.5 ± 12.6	18.9 ± 12.8	18.3 ± 11.8	17.7 ± 9.6	<0.001
FBG, mmol/L	5.9 ± 1.3	6.1 ± 1.4	6.3 ± 1.9	6.3 ± 1.8	<0.001
Triglyceride, mmol/L	1.4 ± 1.2	1.8 ± 1.2	1.9 ± 1.3	1.9 ± 1.3	<0.001
HDL-C, mmol/L	1.6 ± 0.4	1.5 ± 0.4	1.4 ± 0.4	1.4 ± 0.4	<0.001
LDL-C, mmol/L	2.7 ± 0.8	2.9 ± 0.8	3.0 ± 0.8	3.1 ± 0.8	<0.001

Data are the mean ± SD or median (interquartile range), or number (percentage).

WC, waist circumference; BMI, body mass index; SBP, systolic blood pressure; DBP, diastolic blood pressure; Hcy, Homocysteine; FPG, fasting plasma glucose; HDL-C, high-density lipoprotein cholesterol; LDL-C, low-density lipoprotein cholesterol.

### Association between WWI and baPWV in total population

**Figure 1:**
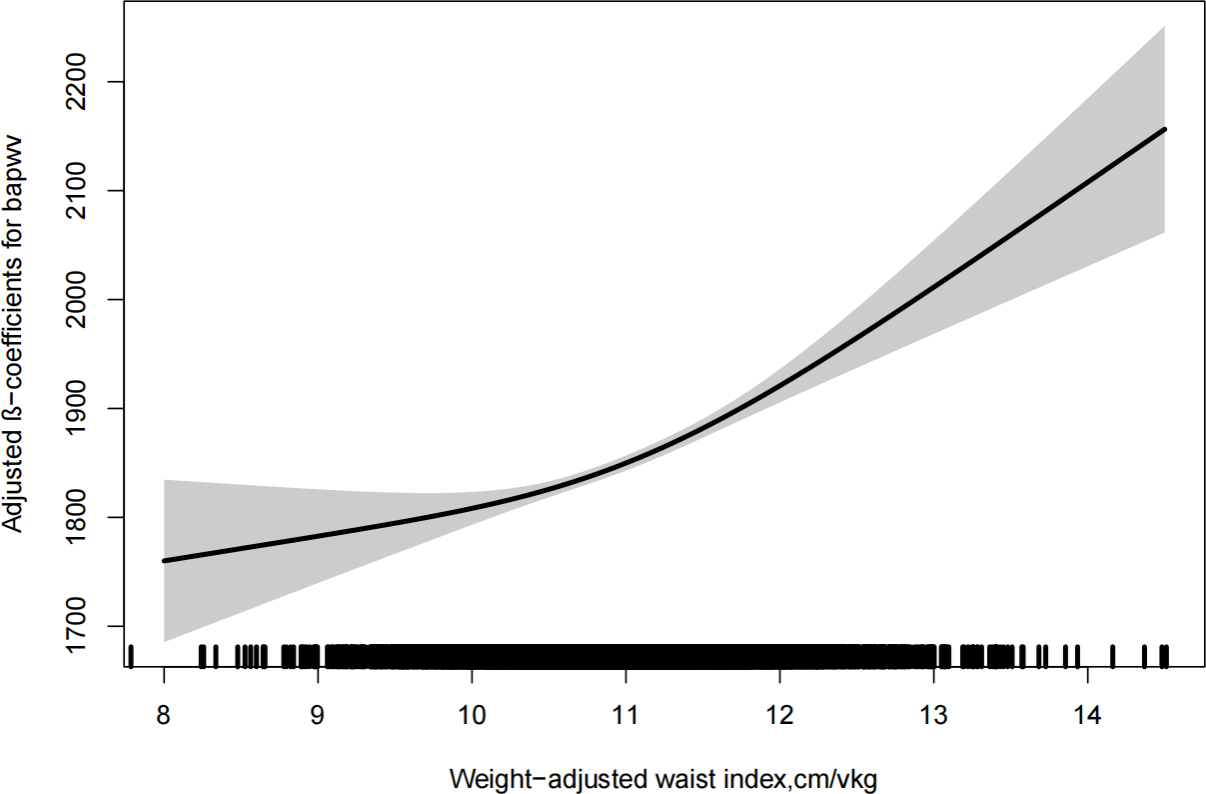
Dose-response relationship between weight-adjusted waist index and baPWV. Models were adjusted for age, sex, current smoking, current drinking, physical activity, BMI, SBP, DBP, duration of hypertension, diabetes mellitus, hyperlipidemia, antihypertensive agents, antidiabetes agents, lipidlowering agents, FPG, homocysteine, triglyceride, HDL-C and LDL-C.

As shown in **Figure 1**, there was a significantly positive association between WWI and baPWV. Moreover, the significant relationship between WWI with baPWV was observed in **Table 2**. After correction for different confounding factors, the positive associations between WWI and baPWV were found in three models (*P* for trend <0.001). For a 1-unit increase in WWI, the baPWV is changed in 57.98mm/s (95% CI 44.06-71.90) in the fully adjusted model. The subjects were stratified into four groups by the quartile value of WWI, comparing the Q1 (<10.5 cm/√kg), the adjusted β of Q2 (≥10.5, <10.9), Q3 (≥10.9, <11.5), and Q4 (≥11.5)were 30.07 (95% CI 3.53-56.62), 67.49 (95% CI 39.59-95.38), 113.65 (95% CI 83.46-143.84) in the model 3.

**Table 2:** Association between weight-adjusted waist index and baPWV in different models.

Weight-adjusted waist index, cm/√kg		Model 1	Model 2	Model 3
		β (95% CI)	β (95% CI)	β (95% CI)
Per 1 unit increase	5232	59.69 (46.26, 73.13)	72.76 (57.43, 88.09)	57.98 (44.06, 71.90)
Q1 (<10.5)	1308	Reference	Reference	Reference
Q2 (≥10.5, <10.9)	1308	24.16 (-4.85, 53.17)	43.36 (13.76, 72.97)	30.07 (3.53, 56.62)
Q3 (≥10.9, <11.5)	1308	65.85 (36.80, 94.90)	91.69 (60.85, 122.53)	67.49 (39.59, 95.38)
Q4 (≥11.5)	1308	111.47 (82.08, 140.86)	137.83 (104.51, 171.14)	113.65 (83.46, 143.84)
*P* for trend		<0.001	<0.001	<0.001

Model 1: adjusted for age.

Model 2: adjusted for age, sex, current smoking, current drinking, physical activity, BMI.

Model 3: adjusted for sex, age, current smoking, current drinking, physical activity, BMI, SBP, DBP, duration of hypertension, diabetes mellitus, hyperlipidemia, antihypertensive agents, antidiabetes agents, lipid-lowering agents, FPG, Hcy, triglyceride, HDL-C and LDL-C.

### Association between WWI and baPWV in different BMI groups

**Figure 2:**
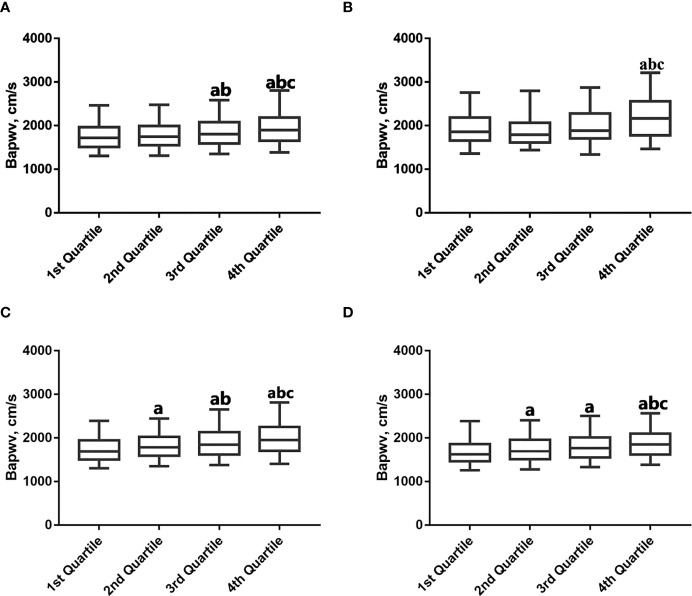
Mean baPWV by weight-adjusted waist index quartiles according to BMI category. **(A)** Total; **(B)** BMI (<18.5kg/m2); **(C)** BMI (18.5-23.9kg/m2); **(D)** BMI (≥24kg/m2). a p < 0.05 compared with the 1st Quartile. b p < 0.05 compared with the 2nd Quartile. c p < 0.05 compared with the 3rd Quartile.

As shown in **Table 3**, the positive associations between WWI and baPWV were maintained in different categories of BMI, even in the normal BMI group (18.5-23.9kg/m^2^). In the normal BMI group, per 1-unit increase in WWI, the baPWV increases by 74.21mm/s (95% CI 54.57-93.85) in model 3. Moreover, the baPWV values increased as the quartiles of WWI (*P* for trend<0.05). Consistently, the same associations between WWI and baPWV were observed among underweight (BMI <18.5) and overweight (BMI ≥24) subjects. **Figure 2** shows the levels of baPWV by quartiles of WWI in total and different BMI populations. The levels of baPWV showed an increasing trend across quartiles of WWI in total and different BMI populations, but this trend was not evident in the underweight group.

**Table 3:** Association between weight-adjusted waist index and baPWV in different BMI subgroups.

Weight-adjusted waist index, cm/√kg		Model 1	Model 2	Model 3
		β (95% CI)	β (95% CI)	β (95% CI)
BMI (<18.5kg/m2)				
Per 1 unit increase	409	108.88 (53.26, 164.50)	112.78 (53.93, 171.63)	94.30 (39.36, 149.23)
Q1 (<10.5)	102	Reference	Reference	Reference
Q2 (≥10.5, <10.9)	102	-70.62 (-207.69, 66.44)	-76.35 (-213.37, 60.68)	-79.26 (-203.86, 45.33)
Q3 (≥10.9, <11.5)	102	14.93 (-122.81, 152.67)	30.07 (-110.13, 170.27)	44.13 (-83.53, 171.78)
Q4 (≥11.5)	103	201.23 (61.97, 340.50)	205.81 (59.25, 352.37)	163.38 (25.32, 301.44)
*P* for trend		0.003	0.003	0.009
BMI (18.5-23.9kg/m2)				
Per 1 unit increase	2221	90.74 (71.26, 110.22)	85.27 (63.78, 106.75)	74.21 (54.57, 93.85)
Q1 (<10.5)	673	Reference	Reference	Reference
Q2 (≥10.5, <10.9)	671	53.51 (12.20, 94.83)	62.22 (20.34, 104.10)	30.03 (-7.43, 67.50)
Q3 (≥10.9, <11.5)	674	108.16 (66.70, 149.61)	110.46 (67.65, 153.27)	89.22 (50.39, 128.04)
Q4 (≥11.5)	673	169.19 (126.87, 211.51)	156.90 (110.64, 203.16)	126.90 (84.88, 168.92)
*P* for trend		<0.001	<0.001	<0.001
BMI (≥24kg/m2)				
Per 1 unit increase	2101	41.17 (19.06, 63.28)	45.59 (21.87, 69.30)	26.11 (5.22, 47.01)
Q1 (<10.5)	533	Reference	Reference	Reference
Q2 (≥10.5, <10.9)	532	18.21 (-22.49, 58.91)	22.79 (-18.16, 63.75)	16.94 (-18.83, 52.70)
Q3 (≥10.9, <11.5)	533	40.58 (-0.53, 81.70)	47.31 (5.28, 89.33)	20.50 (-16.48, 57.48)
Q4 (≥11.5)	533	73.20 (30.80, 115.61)	82.99 (37.40, 128.58)	58.22 (18.11, 98.32)
*P* for trend		<0.001	<0.001	0.007

Model 1: adjusted for age.

Model 2: adjusted for age, sex, current smoking, current drinking, physical activity.

Model 3: adjusted for age, sex, current smoking, current drinking, physical activity, SBP, DBP, duration of hypertension, diabetes mellitus, hyperlipidemia, antihypertensive agents, antidiabetes agents, lipid-lowering agents, FPG, Hcy, triglyceride, HDL-C and LDL-C.

### Stratification analysis

**Figure 3:**
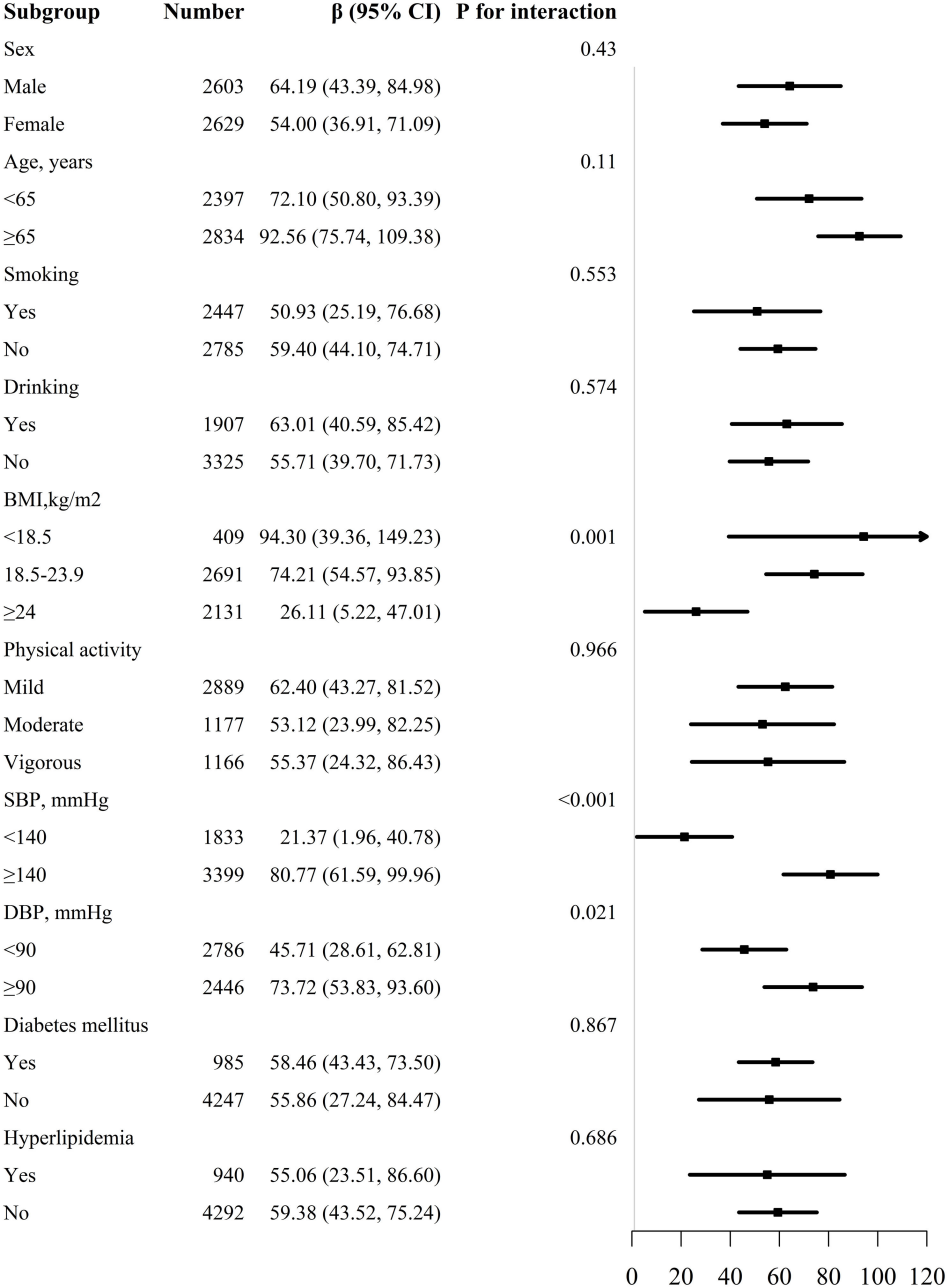
Subgroup analyses of the effect of WWI on baPWV.

We conducted a list of stratified analyses by sex, age (<65 and ≥65 years), BMI (<18.5, 18.5-23.9 and ≥24kg/m^2^), smoking (yes/no), drinking (yes/no), physical activity (mild, moderate and vigorous), SBP (<140 and ≥140mmHg), DBP (<90 and ≥90mmHg), diabetes mellitus (yes/no), and hyperlipidemia (yes/no) as shown in **Figure 3**. We observed that the relationship between WWI and baPWV was consistent in all subgroups except for BMI (*P* for interaction =0.001), SBP (*P* for interaction <0.001), and DBP (*P* for interaction =0.021).

We further divided the population into four groups based on SBP and DBP (group 1: SBP<140mmHg and DBP<90mmHg, group 2: SBP≥140mmHg or DBP≥90mmHg, group 3: SBP≥160mmHg or DBP≥100mmHg, and group 4: SBP≥180mmHg or DBP≥110mmHg), and to explore the association of WWI and baPWV (**Table S1**). Per 1-unit increase in WWI, the baPWV increase by 23.39mm/s (95% CI 2.36-44.42), 72.80mm/s (95% CI 53.84-91.75), 79.03mm/s (95% CI 50.21-107.85), 189.74mm/s (95% CI 96.37-283.11) respectively. A more accentuated increase was observed in the higher BP group.

WWI (cm/√kg) was calculated as WC (cm) divided by the square root of weight (kg). As shown in **Table S2**, significant differences in weight and WC between BMI subgroups. Therefore, there was an interaction between WWI and BMI.

### Sensitivity analyses

As shown in **Table S3A**, the sensitivity analysis was conducted by the exclusion of subjects treated with lipid-lowering agents, and the result was stable. Moreover, we performed a sensitivity analysis restricting the patients treated with lipid-lowering agents, the results remained significant and consistent (**Table S3B**).

### Mediation effect of BP

The unstandardized regression coefficients for the effect of WWI on baPWV without and with SBP and DBP as mediators are shown in **Table 4**. As in **Table 4**, the results indicated that SBP and DBP mediated 28.3% and 8.6% of the relationship of increasing WWI and baPWV, respectively.

**Table 4:** Direct and indirect effects of WWI on markers of Bapwv with blood pressure as mediators in hypertensive patients.

Variables	Total Effect (c)		Direct Effect (c’)		Indirect Effect (ab)		Proportion of Mediation (%)
Mediators and Outcomes	β (95% CI)	*p*-Value	β (95% CI)	*p*-Value	β (95% CI)	*p*-Value	
WWI (cm/√kg)	59.69 (46.26, 73.13)	<0.001					
*via* SBP (mmHg)			42.81 (30.52, 55.10)	<0.001	16.89 (11.19, 22.50)	<0.001	28.3
*via* DBP (mmHg)			55.16 (42.60, 67.71)	<0.001	4.54 (-0.26, 9.34)	0.064	7.6

Regression coefficients c, a, b and c’ are shown in **Figure S1**. All estimates were adjusted for the potential effects of age.

## Discussion

This cross-sectional study evaluated the association between WWI and baPWV in a group of middle-aged and older hypertensive patients. The main findings encompassed the following. We observed that WWI was positively associated with baPWV among patients with hypertension, even though these patients had normal BMI. The association was more significant in patients with higher BP or lower BMI levels. Moreover, this association was stable after excluding the patients treated with lipid-lowering agents. In this relationship, SBP mediated around 28.3% of the total effects. These findings suggest that WWI could be considered as an intervening factor in preventing and treatment of AS, besides BP management.

Numbers of epidemiological studies have demonstrated that obesity was significantly associated with high arterial stiffness (7, 8). A previous meta-analysis of 20 studies showed that modest weight loss was associated with reduced PWV (9). Obesity is usually defined by BMI. Many clinical studies indicated that BMI is positively associated with increased PWV (12, 13). However, several studies suggested that BMI is negatively associated with high arterial stiffness (15, 16). Moreover, Liao et al. conducted a longitudinal study of 1553 subjects found that there was no relationship between the BMI and AS after correction for SBP and DBP (18). There were several reasons for these apparent discrepancies including different study cohorts, ethnic and regional disparity. Furthermore, BMI does not differentiate between lean mass and body fat mass. WWI is an anthropometric index that can be calculated easily. A cross-sectional study suggested that WWI could reflect both body fat and muscle mass (20). In addition, several clinical studies showed that WWI is closely related to an increased risk of diabetes (28), hypertension (24), metabolic syndrome (29), CVD (19), and mortality (30). However, to our knowledge, this is the first study to explore the association between WWI and baPWV in a large population. In this study, it seems that WWI is positively related to baPWV in total and different BMI populations. Moreover, this relationship between 2 was independent of other variables in the multivariate linear regression and mediation analysis.

Notably, the association between WWI and baPWV appeared to be more pronounced in patients with SBP ≥140mmHg or DBP ≥90mmHg. As is well known, hypertension is a major risk for AS (21). Therefore, it is reasonable to postulate that higher BP status may amplify the adverse impact of WWI on baPWV. Our results support this assumption. In stratified analysis by BP, a larger association between WWI and baPWV is observed in patients with higher BP status (**Table S1**).

Several underlying mechanisms might explain the association between WWI and baPWV. WWI was positively associated with fat mass and negatively associated with lean body mass (20). Numbers experimental and human studies suggested that adipocyte hyperplasia and hypertrophy might induce adipokine dysregulation (31). And adipokine dysregulation may lead to vascular inflammation, endothelial dysfunction, and vascular remodeling, resulting in AS (32, 33). Moreover, lower lean mass induces less glucose intake into the muscle and physical activity increases lean mass and improves endothelial function, oxidative stress, insulin resistance, and inflammation, and subsequently improves AS (9). In addition, WWI was an independent risk factor for diabetes (28) and metabolic syndrome (29), all of these diseases can worsen arteriosclerosis.

The strength of our study is that we assess the association between WWI and baPWV in different BMI groups. Moreover, the present study included a large hypertension cohort, subgroup analysis, and mediation analysis. However, this study contains several limitations. First, this study was a cross-sectional study. Hence, the association cannot prove causality. Second, although multivariate correction, it was difficult to exclude any potential confounding effect. Third, the conclusion of the current study applies to the Chinese hypertensive population that may not be directly extrapolated.

## Conclusion

For hypertensive patients, we found that WWI was positively associated with baPWV in different BMI groups. Further longitudinal studies are required to corroborate our findings. Moreover, WWI might be considered as an intervening factor in preventing and treatment of AS, besides BP management.

## Data availability statement

The data that support the findings of this study are available from the corresponding author upon reasonable request. Requests to access these datasets should be directed to XC, xiaoshumenfan126@163.com.

## Ethics statement

The studies involving human participants were reviewed and approved by Ethics Committee of Institute of Bio-medicine, Anhui Medical University and the Second Affiliated Hospital of Nanchang University. The patients/participants provided their written informed consent to participate in this study.

## Author contributions

YX, XH, HB and XC conceived and designed the study. YX, XH, WZ and were involved in the acquisition of data. YX and CY analyzed the data. YX wrote this paper. HB and XC contributed to the revision of manuscript for important intellectual content. All authors contributed to the article and approved the submitted version.

## Supplementary material

Supplementary Figure 1 Study flow diagram.

Supplementary Figure 2 Represent a single mediator model used to test the association between WWI and Bapwv, SBP and DBP as mediators. **(A)** Path c, represents the simple total effect of WWI on baPWV, without adjusting mediators; **(B)** Path c, represents the direct (Path c’) and indirect effect (product of path a and b, ab) of WWI on Bapwv after controlling for the effect of the mediator.
