# Supplementary figures and images for: Association between weight-adjusted waist index and arterial stiffness in hypertensive patients: The China H-type hypertension registry study
Source: Front Endocrinol (Lausanne). 2023 Mar 17;14:1134065. doi: 10.3389/fendo.2023.1134065 (PMC10064138; doi:10.3389/fendo.2023.1134065)

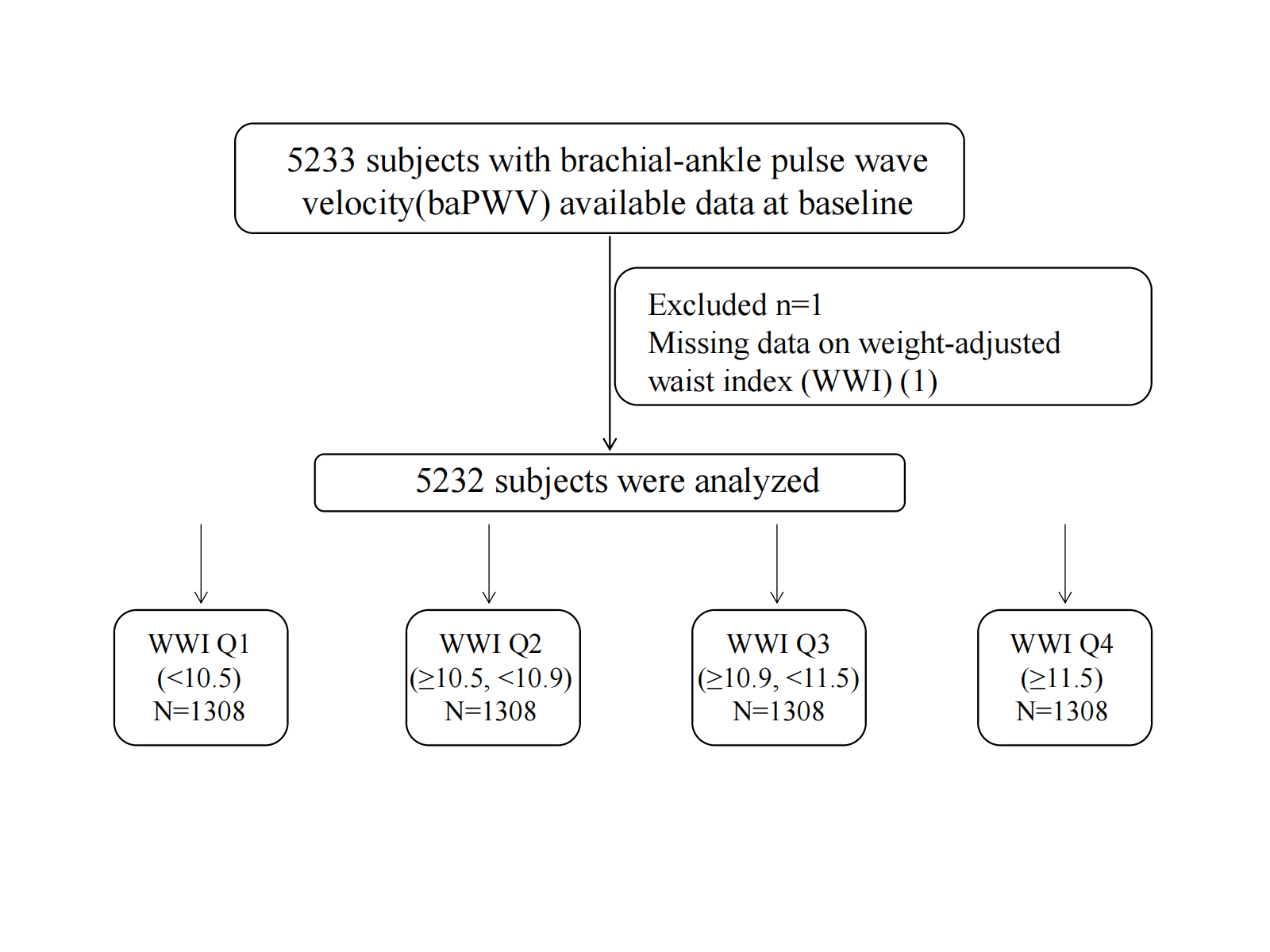

Supplement: Supplementary Figure 1 — Study flow diagram. [file Image_1.tif]

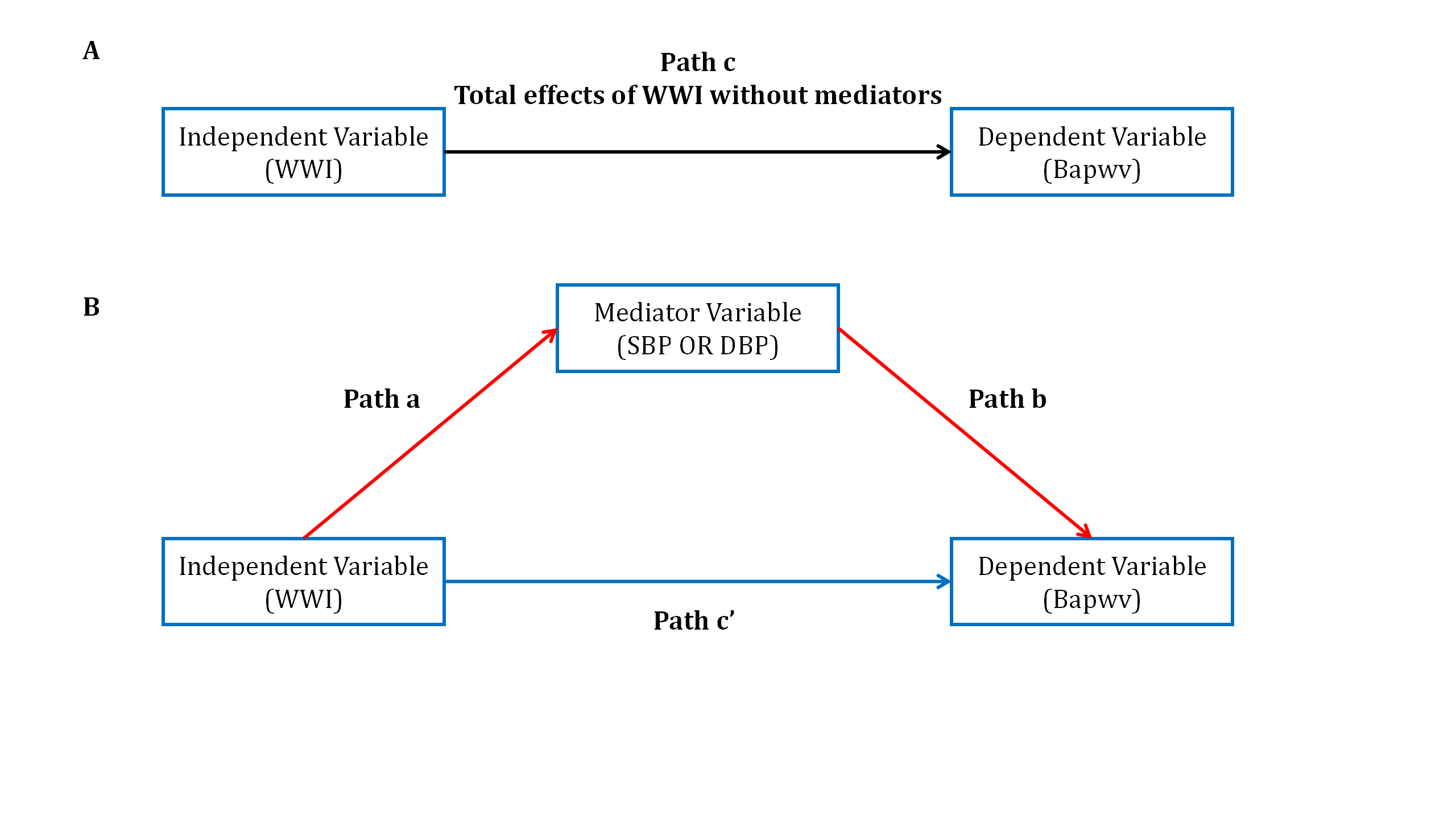

Supplement: Supplementary Figure 2 — Represent a single mediator model used to test the association between WWI and Bapwv, SBP and DBP as mediators. (A) Path c, represents the simple total effect of WWI on baPWV, without adjusting mediators; (B) Path c, represents the direct (Path c’) and indirect effect (product of path a and b, ab) of WWI on Bapwv after controlling for the effect of the mediator. [file Image_2.tif]
