# Supplementary material for: Association between weight-adjusted waist index and arterial stiffness in hypertensive patients: The China H-type hypertension registry study
Source: Front Endocrinol (Lausanne). 2023 Mar 17;14:1134065. doi: 10.3389/fendo.2023.1134065 (PMC10064138; doi:10.3389/fendo.2023.1134065)
Supplement: Supplementary file 3 [file Table_1.doc]

Table S1. Association between WWI and Bapwv stratified by SBP and DBP.

|  | Adjusted Model | | *P* for  interaction |
| --- | --- | --- | --- |
|  | β (95% CI) | *p*-Value |
| SBP<140mmHg and DBP<90mmHg | 23.39 (2.36, 44.42) | 0.029 | <0.001 |
| SBP≥140mmHg or DBP≥90mmHg | 72.80 (53.84, 91.75) | <0.001 |  |
| SBP≥160mmHg or DBP≥100mmHg | 79.03 (50.21, 107.85) | <0.001 |  |
| SBP≥180mmHg or DBP≥110mmHg | 189.74 (96.37, 283.11) | <0.001 |  |

Model was adjusted for age and BMI.
