# Supplementary material for: Association between weight-adjusted waist index and arterial stiffness in hypertensive patients: The China H-type hypertension registry study
Source: Front Endocrinol (Lausanne). 2023 Mar 17;14:1134065. doi: 10.3389/fendo.2023.1134065 (PMC10064138; doi:10.3389/fendo.2023.1134065)
Supplement: Supplementary file 4 [file Table_2.docx]

Table S2. Weight and WC levels in different BMI group.

|  | body mass index, kg/m^2^ | | | P value |
| --- | --- | --- | --- | --- |
|  | <18.5 | 18.5-23.9 | ≥24 |  |
| Weight, kg | 41.8 ± 4.9 | 52.3 ± 6.7 | 65.2 ± 8.6 | <0.001 |
| WC, cm | 67.7 ± 6.1 | 78.2 ± 6.2 | 90.1 ± 6.7 | <0.001 |
| WWI, Q1 (<10.5cm/√kg) | | | | |
| Weight, kg | 42.7 ± 4.7 | 53.6 ± 6.4 | 68.8 ± 8.7 | <0.001 |
| WC, cm | 64.4 ± 4.1 | 73.3 ± 5.0 | 83.6 ± 6.2 | <0.001 |
| WWI, Q2 (≥10.5, <10.9cm/√kg) | | | | |
| Weight, kg | 41.1 ± 4.9 | 53.7 ± 6.6 | 67.8 ± 8.3 | <0.001 |
| WC, cm | 68.4 ± 4.1 | 78.3 ± 4.9 | 88.2 ± 5.4 | <0.001 |
| WWI, Q3 (≥10.9, <11.5cm/√kg) | | | | |
| Weight, kg | 42.1 ± 4.0 | 52.2 ± 6.5 | 65.1 ± 7.7 | <0.001 |
| WC, cm | 72.4 ± 3.4 | 80.5 ± 5.1 | 90.2 ± 5.3 | <0.001 |
| WWI, Q4 (≥11.5cm/√kg) | | | | |
| Weight, kg | 41.8 ± 4.9 | 52.3 ± 6.7 | 65.2 ± 8.6 | <0.001 |
| WC, cm | 67.7 ± 6.1 | 78.2 ± 6.2 | 90.1 ± 6.7 | <0.001 |

WC waist circumference.
