# Supplementary material for: Association between weight-adjusted waist index and arterial stiffness in hypertensive patients: The China H-type hypertension registry study
Source: Front Endocrinol (Lausanne). 2023 Mar 17;14:1134065. doi: 10.3389/fendo.2023.1134065 (PMC10064138; doi:10.3389/fendo.2023.1134065)
Supplement: Supplementary file 5 [file Table_3.doc]

Table S3.A. The sensitive analysis results of patients without lipid-lowering agents.

| Weight-adjusted waist index, cm/√kg | Model 1 | Model 2 | Model 3 |
| --- | --- | --- | --- |
|  | β (95% CI) | β (95% CI) | β (95% CI) |
| Per 1 unit increase | 60.02 (46.28, 73.76) | 72.76 (57.43, 88.09) | 57.98 (44.06, 71.90) |
| Q1 (<10.5) | Reference | Reference | Reference |
| Q2 (≥10.5, <10.9) | 21.92 (-7.71, 51.55) | 41.22 (10.98, 71.47) | 28.04 (0.88, 55.20) |
| Q3 (≥10.9, <11.5) | 64.68 (34.97, 94.39) | 90.32 (58.77, 121.87) | 65.87 (37.29, 94.44) |
| Q4 (≥11.5） | 110.90 (80.85, 140.96) | 137.21 (103.12, 171.30) | 113.14 (82.20, 144.08) |
| *P* for trend | <0.001 | <0.001 | <0.001 |

Model 1: adjusted for age

Model 2: adjusted for age, sex, current smoking, current drinking, physical activity, BMI

Model 3: adjusted for age, sex, current smoking, current drinking, physical activity, BMI, SBP, DBP, duration of hypertension, diabetes mellitus, hyperlipidemia, antihypertensive agents, antidiabetes agents, FPG, triglyceride, HDL-C and LDL-C.

Table S3.B. The sensitive analysis results of patients treated with lipid-lowering agents.

| Weight-adjusted waist index, cm/√kg | Model 1 | Model 2 | Model 3 |
| --- | --- | --- | --- |
|  | β (95% CI) | β (95% CI) | β (95% CI) |
| Per 1 unit increase | 58.79 (-0.87, 118.45) | 63.61 (-8.34, 135.56) | 73.34 (9.02, 137.65) |
| Q1 (<10.5) | Reference | Reference | Reference |
| Q2 (≥10.5, <10.9) | 103.55 (-33.19, 240.29) | 105.40 (-38.82, 249.63) | 119.35 (-6.98, 245.67) |
| Q3 (≥10.9, <11.5) | 125.71 (-5.73, 257.15) | 137.04 (-6.67, 280.76) | 129.41 (3.11, 255.70) |
| Q4 (≥11.5） | 148.86 (15.83, 281.89) | 158.51 (4.28, 312.74) | 186.88 (47.90, 325.87) |
| *P* for trend | 0.034 | 0.048 | 0.013 |

Model 1: crude model

Model 2: adjusted for sex, age, current smoking, current drinking, physical activity, BMI

Model 3: adjusted for sex, age, current smoking, current drinking, physical activity, BMI, SBP, DBP, duration of hypertension, diabetes mellitus, hyperlipidemia, antihypertensive agents, antidiabetes agents, FPG, triglyceride, HDL-C and LDL-C.
